# Emotional Distress Following Childbirth: An Intervention to Buffer Depressive and PTSD Symptoms

**DOI:** 10.5964/ejop.v11i2.779

**Published:** 2015-05-29

**Authors:** Paola Di Blasio, Sarah Miragoli, Elena Camisasca, Angela Maria Di Vita, Rosalia Pizzo, Laura Pipitone

**Affiliations:** a Catholic University of Milan, Milan, Italy; b e-Campus University, Novedrate, Italy; c Juvenile Court of Palermo, Palermo, Italy; d University of Palermo, Palermo, Italy; Warren Alpert Medical School, Brown University, Providence, USA; Department of Psychology, University of Toledo, Toledo, Spain

**Keywords:** intervention, childbirth, depression, Post-Traumatic Stress Disorder

## Abstract

Childbirth for some women is a negative experience associated with depressive and post-traumatic symptoms. The preventive actions focusing on helping mothers to cope with negative emotions experienced after childbirth are strongly recommended. It is also recommended both to intervene early and on all women to avoid the risk that these symptoms can worsen in the months after childbirth. The intervention described in the current study is focalized on the elaboration of post-partum negative thoughts and emotion through a writing task, with the purpose to help new mothers to reflect, understand, evaluate and, thus, reformulate the stressful situation with new beliefs and emotions. 176 women aged from 19 to 43 years (M = 31.55, SD = 4.58) were assessed for depression and PTSD in the prenatal phase (T1). In about 96 hours after childbirth they were randomly assigned to either “Making Sense condition” (MS: in which they wrote about the thoughts and emotions connected with delivery and childbirth) or “Control-Neutral condition” (NC: in which they wrote about the daily events in behavioural terms) and then reassessed for depression and PTSD (T2). A follow up was conducted 3 months later (T3) to verify depression and posttraumatic symptoms. The results showed that depressive symptoms decreased both at 96 hours and at 3 months as a result of making-sense task. Regarding the posttraumatic symptoms the positive effect emerged at three months and not at 96 hours after birth.

For some women the birth of a child is a critical key life transition associated with various symptoms. Of all the perinatal affective disorders, maternity blues and depression are the most commonly known and, in research studies in the last few years, also Post-Traumatic Stress Disorder (PTSD) is beginning to be recognized as a relevant symptomatic disease. Maternity blues (MB) occurs in the first few days after delivery with estimates of prevalence ranging from 30% to 75% (O’Hara & Swain, 1996). It is a transitory psychological disorder, characterized by mild depressive symptoms, tearfulness, sorrow/weeping, unstable moods, anxiety, and confusion (Newport, Hostetter, Arnold, & Stowe, 2002). This disturbance tends to disappear in many women within a couple of weeks. However, about 20% of women suffering from MB are diagnosed as having major depression in the first year following delivery (Campbell, Cohn, Flanagan, Popper, & Meyers, 1992; O’Hara, Schlechte, Lewis, & Wright, 1991). Some research, moreover, confirmed the stability of the symptoms in time, considering that the maternal mood at one week can predict the postnatal depression at four and eight weeks, because of the persistence of the same risk factors (Dennis, Janssen, & Singer, 2004). The major risk factors for developing postnatal depression (PND), defined as an episode of depression within a year after the birth of a child, include depression and anxiety during pregnancy, postpartum MB and past history of psychiatric illness. The occurrence of stressful life events during pregnancy and the absence of social support networks increase a mother’s risk of developing PND (Beck, 2001; O’Hara & Swain, 1996; Robertson, Grace, Wallington, & Stewart, 2004). The PND affects a significant number of women with prevalence rate about 19.2% in the first three months and 12.9% in the first year from birth (Gaynes et al., 2005), with a mean estimated of about 13% in prenatal and postnatal phases (Bennett, Einarson, Taddio, Koren, & Einarson, 2004).

For some women childbirth can be, also, a critical or a severe traumatogenic event contributing to poor psychological adjustment. Posttraumatic stress essentially involves three distinct clusters of symptoms: re-experiencing the event (flashbacks), persistent avoidance of any stimuli associated with the event, and increased arousal. In childbirth experiences, named atypical or “irregular” (for reviews: Andersen, Melvaer, Videbech, Lamont, & Joergensen, 2012; Olde, van der Hart, Kleber, & Van Son, 2006), characterized by risk factors as serious prematurity, health difficulties in the mother or/and the child and stillbirth, a prevalence rate of full PTSD is between 20%-25% and partial symptoms between 26% and 41%. Also regular childbirth could be traumatic and, in this context, two principal factors are relevant: subjective emotional distress alone and subjective distress associated with others problems, above all depression. The prevalence rate of full PTSD in regular childbirth without medical complication ranges from 2.8% to 5.6% at six weeks post-partum (Ayers & Pickering, 2001; Creedy, Shochet, & Horsfall, 2000; Czarnocka & Slade, 2000; Wijma, Soderquist, & Wijma, 1997) and then decreases to 1.5% at six months after childbirth (Ayers & Pickering, 2001; Wijma, Soderquist, & Wijma, 1997). Moreover, approximately between 21.3% and 33% of women may experience the birth itself as traumatic with three or more traumatic stress symptoms at 4-8 weeks post-partum (Creedy, Shochet, & Horsfall, 2000; Davies, Slade, Wright, & Stewart, 2008; Soet, Brack, & Dilorio, 2003). Elmir and colleagues (2010) in a qualitative review state that the negative emotions (fear, pain, helplessness, threat for one’s own life and the life of the baby, negative appraisal of the delivery, fear of losing control, *etc.*) explain well a large spectrum of problems connected with childbirth. The fear of childbirth was judge so relevant problem that in Finland, Salmela-Aro and colleagues (2012) promoted among nulliparous pregnant women with an intense fear of childbirth, a preventive psycho-educative group therapy intervention, based on social cognitive theories on behavioral control and individual coping resilience, which was effective in increasing the mothers’ preparedness for childbirth and which was predictive of an increase in positive motherhood. Negative feelings of birth could also be associated with other problems and, among these, depression is the strongest predictive variable of the full PTSD symptoms (Denis, Parant, & Callahan, 2011). Epidemiological research found a high degree of co-morbidity between depression and PTSD (Davidson & Fairbank, 1993) and a cohort study by Seng and colleagues (2013) confirms that PTSD and depression symptoms overlap, and PTSD that is chronic and severe often is comorbid with depression.

In summary, postnatal depression and PTSD are serious mental health problems and for these reason, in all studies the preventive actions focusing on helping mothers to cope with negative emotions experienced after childbirth are strongly recommended. Efforts to prevent post-partum depression have been more common (O’Hara, 2009; O’Hara & McCabe, 2013) than those to prevent PTSD (Lapp, Agbokou, Peretti, & Ferreri, 2010), which is still an underestimated phenomenon. However there is little or mixed evidence for the effectiveness of PND prevention programmes. Two reviews (Dennis, 2005; Dennis & Dowswell, 2013) on fifty-six and on twenty-eight trials, involving almost 17.000 women, treated with psychosocial and psychological interventions (such as antenatal and postnatal classes, professional interventions, home visits, debriefing, and interpersonal psychotherapy) showed that postnatal interventions were more beneficial than those that incorporated an antenatal component. In addition, the women who received a psychosocial or psychological postnatal intervention were significantly less likely to develop postpartum depression compared with those receiving standard care. The more promising interventions are: (1) intensive, individualized postpartum home visits, provided by public health nurses or midwives; (2) lay (peer)-based telephone support; and (3) interpersonal psychotherapy.

The moderate effects of psychological treatments and the equal effectiveness of different types of interventions (cognitive behaviour therapy, interpersonal psychotherapy and counseling, and also social support interventions) emerges both from the Cuijpers, Brännmark, and van Straten (2008) meta-analysis on seventeen controlled and comparative research studies in postpartum depression and from a review of Muñoz, Cuijpers, Smit, Barrera, and Leykin (2010) on major depression, which included several studies with postpartum. Interesting results supporting universal prevention (*i.e.* directed at all the women after childbirth), are reported by Brugha, Morrell, Slade, and Walters (2011), in a prospective randomized cluster trial, on two groups of women with “subthreshold” depression scores (EPDS *<* 12 at six weeks postpartum) followed for eighteen months. Women in the intervention cluster (which provided for person-centred counseling or CBT-based counseling from health visitors) were significantly less likely to become depressed than women in the usual care cluster. This research provides new evidence of a universal, enduring preventive effect for depression in women who screen negative for depression postnatally. The effectiveness of the intervention is explained both by the fact that the focus of care was on the mother’s psychological well-being, not just on the physical welfare of the child, and by the opportunity for mothers to recognize and openly communicate their emotions without the fear of being stigmatised (Brugha et al., 2011).

On the basis of the above studies and the results emerging from Brugha et al. (2011), we decided to verify whether an universal early and “light” intervention of narration of childbirth experience (making sense task), focalized on the elaboration of negative thoughts and emotion, can improve women‘s welfare by opposing the distress symptoms. As McAdams’ (1993, 1996, 2001) studies on narration have demonstrated the identity itself takes the form of a story based on selectively appropriate aspects of biographical facts that make sense in the specific and cultural context. In this sense we think that the writing task about a crucial and transitional phase as a childbirth can favour both the continuation and the synchronically and diachronically integration of identity. We consider, in fact, that childbirth has the peculiarity to be an event both full of expectations, which can turn out to be disappointing and discrepant, and characterized by the co-presence of positive feelings about the child’s birth that are explicitly recognized, shared and self-aware along with negative emotions, neither admitted and recognized nor legitimated, due to the loss of control, pain and solitude, which creates an emotional disequilibrium and the perception of the extraneousness of oneself and of others (Di Blasio, Ionio, & Confalonieri, 2009; Ionio & Di Blasio, 2014).

To deal with the complex emotive condition related to childbirth, we chose to experiment with the efficacy of a cognitive-emotional restructuring and re-signification intervention of the childbirth experience, with the purpose to help new mothers, through the narrative, to reflect, understand, evaluate, and thus reformulate the stressful situation with new beliefs directed to coherent objectives (Park, 2010; Park & Ai, 2006). This research hypothesis refers to the “Meaning and Meaning Making Model” which has received great attention in the last few years and has been applied to stressful life experiences in various contexts, among which health and clinical psychology. Some research on bereavement and cancer survivors have reported that meaning-making is related to better adjustment, compared with not having to search for meaning. The cross sectional study by Tolstikova, Fleming, and Chartier (2005), on seventy-two women and twelve men who lost their love ones in motor vehicle accident, showed that the intensity of grief was significantly related to symptoms of trauma (avoidance, intrusive, dissociative experience, anxiety, and depressive mood) and that individual who failed to find meaning had greater intensity of grief feelings and more extensive trauma symptoms. In sample of one hundred ninety-one men and eleven women affected by life-limiting cardiac illness, meaning making was strongly related to better physical and mental quality of live, which in turn is important to reduce re-hospitalization and mortality (Park, Malone, Suresh, Bliss, & Rosen, 2008)

“Meaning” can be defined as the “mental representation of possible relationships among things, events, and relationships which connects things” (Park, 2010, p. 257). Situational meaning, in particular, refers to meaning in the specific context and describes an ongoing set of processes and outcomes, including assignment of meaning to the event (appraised meaning), determination of discrepancies between appraised and global meaning, meaning making, meanings made, and adjustment to the event (Park, 2010, p. 259). According to the Meaning Making Model, the cognitive and emotional discrepancy between the appraised meaning of the event and the general individual’s global beliefs and expectations, underlies the level of distress experienced. The childbirth experience could be characterized precisely by this. In fact, the negative distress observed in many women after childbirth (see reviews: Andersen et al., 2012; Elmir, Schmied, Wilkes, & Jackson, 2010) can be explained as the effect of a discrepant psychological condition due to both the concrete childbirth experience, more demanding, painful, long and stressful than expected, and to the simultaneous presence of the negative and positive emotions, described above. The purpose of intervention is to seek, through elaboration, to reduce this discrepancy between expectations and contrasting emotions since this could be a precursor to developing a coherent story of the stressful events which is also associated with better outcomes (Boals, 2012; Ramírez-Esparza & Pennebaker, 2006).

## Objectives and Hypotheses

The objective of this study, based on a universal prevention intervention, is to verify whether giving meaning to stressful experience of childbirth can moderate the association between the antenatal depressive and post-traumatic symptoms (T1) and the same postnatal symptoms (T2 and T3). In particular, the first research hypothesis (H1) is that the making-sense task enables the reduction of depressive and post-traumatic (re-experiencing, avoidance, and hyperarousal) symptoms immediately after childbirth (96 hours; T2): more precisely, we expect that women assigned to the “making-sense group” (asked to write about childbirth) will show a significant reduction of depressive and post-traumatic symptoms, while women assigned to the “control-neutral group” (asked to write about neutral and behavioural events) have an increase in these symptoms or do not present differences compared to antenatal phase. We consider, in fact, that a making-sense task would set off reflective and restructuring mechanisms on childbirth experience, with a containing effect on the symptoms at 96 hours and we also think that such a result could have a protective value by reducing the risk that the distress can worsen in the three post-partum months.

The second research hypothesis (H2) is that this positive effect lasts in time and that at the follow up at 3 months (T3) the women assigned to the “making-sense group” will present a significant reduction of depressive and post-traumatic symptoms, while women assigned to the “control-neutral group” (asked to write about neutral and behavioural events) will have an increase in these symptoms or do not present differences compared to antenatal phase.

## Method

### Participants

Two hundred and twelve women between the 32nd and 40th weeks of pregnancy were recruited during the prenatal courses and at the hospitals (Time 1, T1) of two large Italian cities in the North and in the South (Milano and Palermo). The researchers explained individually to each pregnant woman the research protocol approved by the appropriate institutional review board and obtained their spontaneous and unpaid consent to participate in the study. The informed consent contained the description of all the phases of the research and of the instruments. Regarding the making-sense task the participants were informed that, after childbirth, they would receive an envelope with the material necessary to carry out a writing task on events in her life. The difference between the two writing tasks (meaning making-task *vs*. neutral) has not been made explicit, to avoid an effect of influencing. The inclusion criteria to participate in the research were: an adequate knowledge of the Italian language in order to understand the questionnaires, no psychiatric diagnosis in anamnesis, and age from eighteen years old upwards.

176 women out of 212 (refusal rate: 16.98%), aged 19-43 years (*M* = 31.55, *SD* = 4.58), participated in all three phases of the study. Most of them were married or cohabiting (84.1%) and had a medium-high level of education: 2.2% with a PhD (nineteen years of education), 35.8% with a degree (seventeen years), 46.6% with a high-school certificate (thirteen years), 15.4% with a middle-school certificate (eight years). The majority were employed (78.4%) and of Italian nationality (96%). The new-born were 51.1% female and 48.9% male, and most of the women were primiparous (61.9%). As regards the obstetric characteristics, 61.9% of the women participated in the antenatal course and the majority had full term pregnancies: 24.4% had a spontaneous natural childbirth without epidural analgesia, 50% spontaneous with epidural analgesia, 8% planned caesarean and 17.6% emergency caesarean. At birth, most of the babies were healthy: in fact in 89.2% the Apgar score at minute one after delivery was normal, in 2.5% was moderately depressed, and in 8.3% of babies the data were missing.

Thanks to cooperation with medical and nursing staff, the women were re-contacted in the first week after childbirth (Time 2, T2) to effect the second research phase. In this phase the 176 women were randomly assigned at one of the two groups: “Making Sense condition” (MS) and “Control-Neutral condition” (NC). The MS group was composed by 87 women, ranged from 22 to 43 year (*M* = 32.10, *SD* = 4.37), and the NC group by 89 women, ranged from 19 to 39 year (*M* = 31.00, *SD* = 4.77). The comparison between the two groups indicates no significant differences in age (*t* (174) = 1.60, *p* = .11), marital status (χ^2^(1) = 1.37, *p* = .30), parity (χ^2^(3) = 2.91, *p* = .41), baby gender (χ^2^(1) = .208, *p* = .18), participation in antenatal courses (χ^2^(1) = .65, *p* = .72), and birth condition (χ^2^(3) = 4.29, *p* = .23). Three-four months later (Time 3, T3) researchers re-opened the contacts and, by appointment, conducted a telephonic follow-up interview.

### Procedure

The first phase of this study took place during antenatal courses or medical checks between the 32nd and 40th week of pregnancy (T1). After each woman provided informed consent for her participation in the three phases of the research, she completed the questionnaires assessing demographic characteristics, depression (*Beck Depression Inventory II*, BDI; Beck, Steer, & Brown, 1996) and PTSD symptoms (*Los Angeles Symptoms Checklist*, LASC; King, King, Leskin, & Foy, 1995). The second phase took place in at 3-4 days post-partum (T2). In about seventy-two hours, after having verified the absence of severe complications connected to labour and established the good health of mother and child, each participant was randomly assigned to either the “Making Sense condition” (MS), in which she wrote about the thoughts and emotions connected with delivery and childbirth, or to the “Neutral-Control condition” (NC), in which she wrote about her daily event in behavioural terms. The content of making sense intervention was partially derived from Pennebaker’s expressive writing paradigm (Pennebaker, 1997). The instructions for the MS group were to write about deep emotions, expectations, and thoughts connected with delivery and childbirth, describing also the most secret feelings and thoughts which they have not told, nor would tell, to anyone. The women of the NC group were asked to describe the daily events, referring only to events and behaviours without mentioning thoughts and emotions. The instructions were given in a sealed envelope in which the women put their tasks. The writing session was about twenty minutes in duration. Compared with Pennebaker’s (1997) standard protocol, which recommended writing three or four essays, the participants of this study wrote on only one occasion. Although Pennebaker and the results of the more important meta-analyses (Frattaroli, 2006; Frisina, Borod, & Lepore, 2004; Smyth, 1998) found that to improve mental and physical health benefits multiple writing occasions are needed, we here opted for a modified and simplified protocol, which we called “making sense”, to distinguish it from Pennebaker’s standard protocol. The reason for this choice is that more writing sessions would have risked being too demanding for the new mothers, who were already weakened by the childbirth, occupied with the contact with the new-born, undergoing various medical checks and stressed by parents’ and friends’ visits. The decision to carry out the making-sense task in the first days after childbirth was another specific choice to intervene early and preventively on widespread and acute symptoms not yet chronicized and stabilized. In addition, coherently with the aims of this research, the purpose was to carry out a light intervention of making sense of negative and unexpected emotions and thoughts aroused by childbirth and not to verify in a strict sense the efficacy of the expressive writing standard procedure. At one-two days from the restitution of the written account (*M* = 96 hours after childbirth), all the participants completed the BDI and the PPQ (*Perinatal PTSD Questionnaire*; Callahan, Borja, & Hynan, 2006) to evaluate respectively the depression and the PTSD. In the third phase of the research, at three months after childbirth (T3), the women were re-assessed with the BDI and the PPQ, through a telephone interview on the day and at the time of their choice.

### Measures

#### Depression

To assess depression symptoms the *Beck Depression Inventory-II* (BDI; Beck et al., 1996; Italian validation by Ghisi, Flebus, Montano, Sanavio, & Sica, 2006), was administered (at T1, T2, and T3). It includes 21 items asking about cognitive, motivational, affective and behavioural symptoms of depression. Each item was scored on a 4-point scale with a total score of 63. Based on the Italian validation, a cut-off score ≥ 12 identify the presence or the absence of depression. We considered the scores of 13-19: mild depression; 20-28: moderate depression; and 29-63: severe depression.

#### Post-Traumatic Stress Disorder

To assess post-traumatic stress symptoms two measures were employed: the *Los Angeles Symptom Checklist* (LASC; King, King, Leskin, & Foy, 1995), suitable for the general post-traumatic symptoms was used in the prenatal phase (T1); and the modified version of *Perinatal PTSD Questionnaire* (PPQ; Callahan, Borja, & Hynan, 2006), suitable for the specific symptoms connected to childbirth, was used in the two post-natal phases (T2 and T3). *Los Angeles Symptom Checklist* is a self-report measure of 43 items and includes 17 items that address symptoms related to re-experiencing, avoidance and numbing, and hyperarousal PTSD clusters. Each item is made up of a word or phrase evaluated on the five-point Likert scale from 0 (no difficulty) to 4 (very great difficulty). The sum of the 17 items scores (ranged from 0 to 68) permits the evaluation of the PTSD gravity (Severity index) while the sum of all 43 items indicates difficulty in adaptation (Distress index), as a consequence of traumatic experiences. In the present study the Severity index that addresses the “core” PTSD symptoms was used. The LASC cutting score for PTSD Severity index was 25.26 (King et al., 1995, p. 14). In the present study the α coefficient value for the 17 Severity items was .90; a value comparable to those detected across three sample groups by King and colleagues (1995) ranged from .89 to .94.

To assess the specific stress symptoms post-partum, the modified version of *Perinatal PTSD Questionnaire* was administered (T2 and T3). The PPQ specifically developed for routinely identifying distressed mothers who are experiencing perinatal symptoms of post-traumatic distress, it is composed of 14 items on a five-level Likert scale (scored 0 to 4) from 0 point (not at all or never) to 4 points (very often). The first three items describe symptoms of unwanted intrusions, the next six describe symptoms of avoidance or numbing of responsiveness, and the last five items symptoms of hyperarousal. The total possible score on the modified PPQ ranged from 0 to 56. Responses to the questions are summed for a total score. A score of 19 or higher on the PPQ indicates clinically significant distress that merits referral of the mother to a mental health care professional (Callahan & Borja, 2008, p. 54). The PPQ has demonstrated appropriate internal consistency (α from .85 to .90) and test-retest reliabilities (*r* = .92; Callahan & Borja, 2008, p. 53-54). In the present study internal consistency was from .85 to .88.

### Strategy of Analysis

Data analysis was conducted in several steps. Firstly, we presented descriptive statistics regarding the percentages of women with clinical levels of depression and post-traumatic stress in the three research phases. Secondly, a set of regression analyses was conducted to examine whether the making-sense task moderates the relationship between prenatal (T1) and post-natal depressive and post-traumatic (PTSD total score, re-experiencing, avoidance, and hyperarousal) symptoms after 96 hours (T2) and at 3 months (T3). We computed a multiple regression analysis (Baron & Kenny, 1986; Holmbeck, 1997) using the prenatal score, the writing conditions (0 = control group; 1 = making sense), and the interaction products among these variables (Koopman et al., 2005). Given that to evaluate the post-traumatic symptoms, we employed different measures in prenatal (LASC) and in post-natal (PPQ) phases, which give differing scores, we transformed the raw scores into z-scores for all PTSD variables with the purpose of making the results comparable. Regarding the depression scores employed in regression analysis, all independent variables were “centered” to reduce collinearity among the variables.

## Results

### Descriptive Statistics

The descriptive statistics showed that at prenatal phase (T1), 5.68% (*n* = 10) of women present clinical level of PTSD severity (LASC) with a cut-off ≥ 25.26, and 3.9% (*n* = 7) reported levels of moderate and severe depression (BDI) with a cut-off ≥ 20. The check about the randomization of the two groups (see Table 1) “making-sense” (MS) and “neutral-control” (NC) in the prenatal phase (T1) preceding the making-sense task, showed no significant differences in the mean scores for post-traumatic severity (MS: *M* = 10.94, *SD* = 6.35 *vs.* NC: *M* = 12.81, *SD* = 7.51; *t*(174) = -1.77, *p* = .08), while the women in the NC group have a higher scores in depression symptoms than women assigned to MS group (MS: *M* = 9.02, *SD* = 4.28 *vs.* NC: *M* = 10.97, *SD* = 4.99, *t*(174) = -2.76, *p* < .01).

After childbirth (T2) and at 3 months (T3), the percentage of new mothers with clinical post-traumatic symptoms (measured with PPQ, cut off ≥ 19) was respectively 4.6% and 1.1% in MS group and 6.7% and 5.6% in NC group. In the same times (T2 and T3) clinical depression was reported by 1.1% and 0% of women in the MS group and by 4.49% and 5.61% in the NC group.

**Table 1:** Descriptive Statistics for Depression and PTSD Symptoms at Prenatal, 96 Hours and 3 Months After Childbirth

Measures	Prenatal scores (T1)				96 hours scores (T2)				3 months scores (T3)			
	MS group		NC group		MS group		NC group		MS group		NC group	
	*M*	*SD*	*M*	*SD*	*M*	*SD*	*M*	*SD*	*M*	*SD*	*M*	*SD*
Total Depression (BDI)	9.02	4.28	10.97	4.99	7.84	4.58	10.18	5.32	6.74	3.73	10.98	5.90
Total PTSD (z-scores)	-.134	.91	.131	1.07	-.108	.93	.106	1.05	-.339	.70	.331	1.13
PTSD Re-Experiencing (z-scores)	.075	.93	-.073	1.06	-.040	.94	.039	1.72	-.139	.84	.136	1.12
PTSD Avoidance (z-scores)	-.179	.79	.175	1.14	-.067	.99	.066	1.00	-.269	.78	.263	1.11
PTSD Hyper-Arousal (z-scores)	-.128	.96	.126	1.02	-.133	.83	.130	1.09	-.333	.72	.325	1.12

### Outcome of Making-Sense Task

A multiple regression analysis conducted to verify the moderational effect of making-sense task on the association between antenatal depressive and post-traumatic symptoms (T1) and the same postnatal symptoms (T2 and T3).

#### Depression

At first, two distinct regression analyses were performed on the total scores of depression at 96 hours (T2) and at 3 months (T3) after childbirth (see Table 2). Results showed that the models are strongly significant and explained a certain amount of variance (T2: *F*(3,175) = 12.56, *AdjR^2^* = .16, *p* < .001; T3: *F*(3,175) = 24.80, *AdjR*^2^ = .29, *p* < .001).

**Table 2:** Regression Analysis Predicting Depression Scores at 96 Hours (T2) and at 3 Months (T3)

	96 Hours (T2)			3 Months (T3)		
Predictor variables	*B*	*SE B*	β	*B*	*SE B*	β
*Constant*	8.97	.36		8.74	.34	
Prenatal Symptoms Scores	.35	.07	.32**	.37	.07	.33**
Writing Condition	-1.60	.72	-.16*	-3.53	.69	-.33**
Prenatal Symptoms Scores*Writing Condition	-.15	.15	-.07	-.39	.15	-.16*

*Note.* *p < .05. **p < .001.

**Figure 1:**
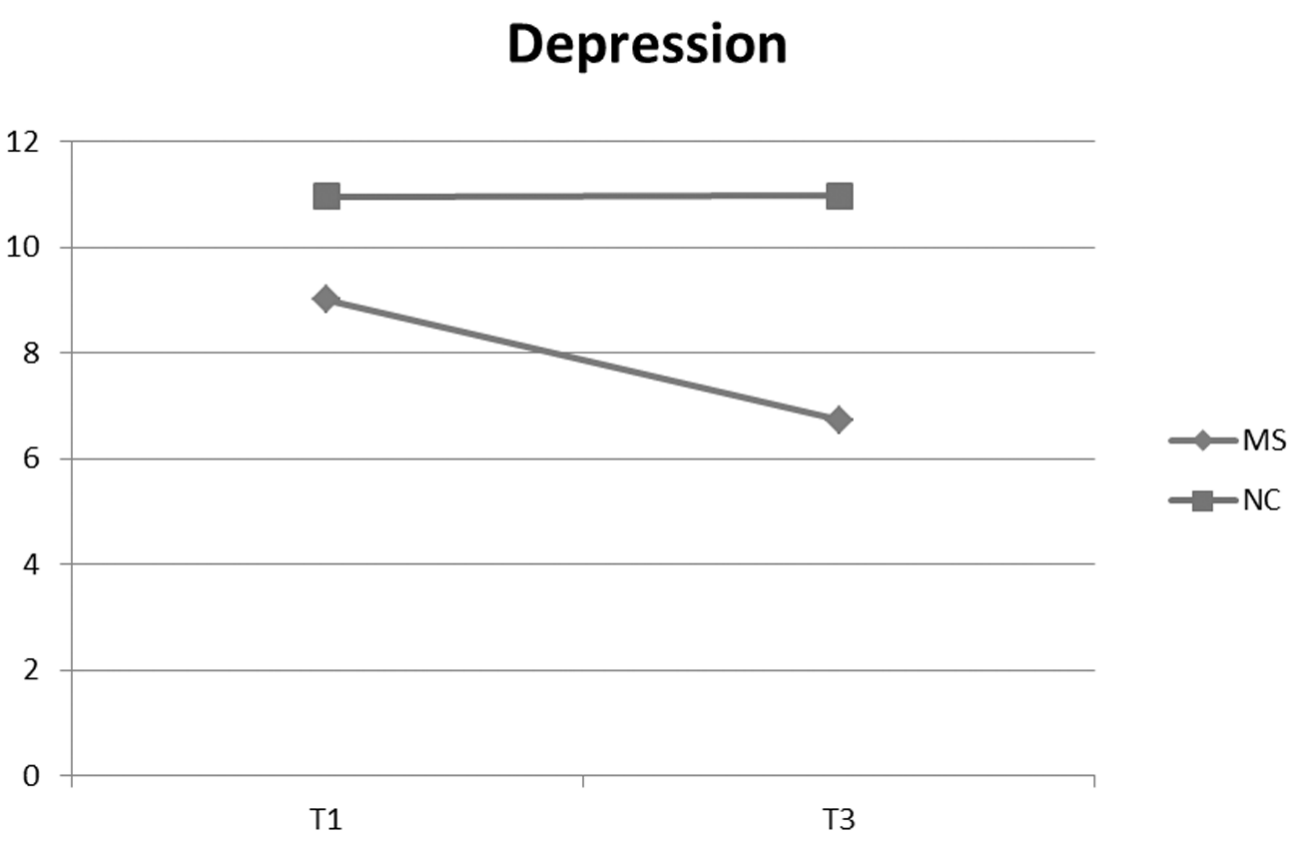
Writing conditions and mean of depression scores at T1 and T3.

At 96 hours (T2), both prenatal depressive symptoms (β = .32) and the writing condition were significant (β = -.16; see Table 2). This result indicated that women assigned to MS group had a greater reduction in depressive symptoms compared to the NC group. After 3 months (T3), besides the significant effect of prenatal symptoms (β = .33) and the MS condition (β = .33), also the interaction product of these variables was significant (β = -.16). The direction of this interaction, verified with two regressions in the MS and NC groups (with independent variable the prenatal depression score and dependent variable the 3 months depression scores), is significant in the NC group (*F*(1,88) = 25.22, *AdjR^2^* = .21, β = .47, *p* < .001), but not in the MS group (*AdjR^2^* = .02, *F*(1,86) = 3.39, β = .19, *p* > .05). Figure 1, in which are pictured the means of depressive symptoms, at prenatal (T1) and at 3 months (T3), show a decrease in the MS group and a substantial stability in the control-neutral group. The dashed line indicates non-significant association.

#### Post-Traumatic Symptoms

As regards post-traumatic symptoms (Total score) the two distinct regression analyses demonstrated that both the model at 96 hours (T2; *F*(3,175) = 11.609, *AdjR^2^* = .154, *p* < .001) and at 3 months (T3; *F*(3,175) = 18.166, *AdjR^2^* = .29, *p* < .001) are significant (see Table 3).

**Table 3:** Regression Analysis Predicting PTSD Scores at 96 Hours (T2) and at 3 Months (T3)

	96 Hours (T2)			3 Months (T3)		
Predictor variables	*B*	*SE B*	β	*B*	*SE B*	β
*Constant*	-.02	.07		-.01	.06	
Prenatal Symptoms Scores	.31	.07	.31**	.38	.06	.38**
Writing Condition	-.18	.14	-.09	-.57	.12	-.28**
Prenatal Symptoms Scores*Writing Condition	-.39	.14	-.08	-.35	.13	-.17*

*Note.* *p < .05. **p < .001.

**Figure 2:**
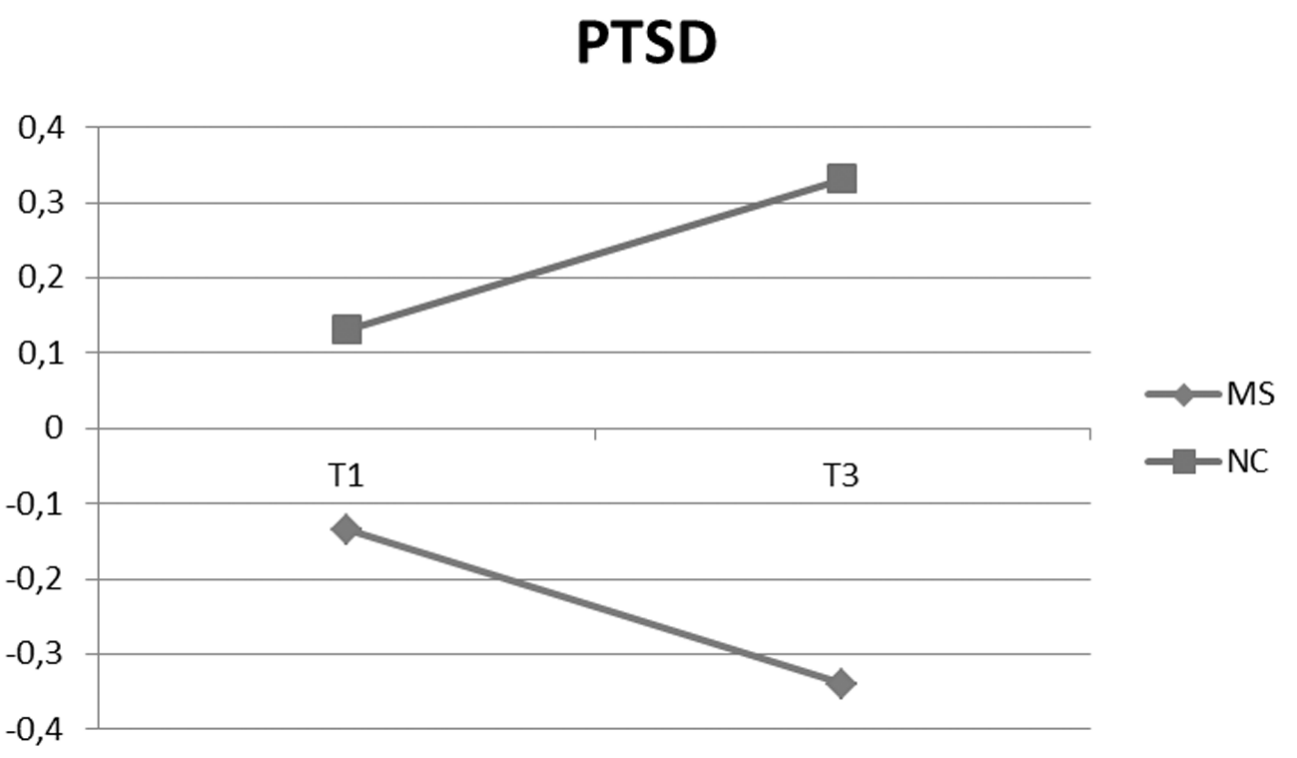
Writing conditions and mean of PTSD total scores (z-Scores) at T1 and T3.

However, unlike the depression, for the post-traumatic symptoms immediately after childbirth (T2) the MS condition compared with NC condition did not show significant effect. At 3 months (T3) both the MS condition (*p* < .001) and the interaction with prenatal symptoms (*p* < .01) are significant. The direction of this interaction, verified with two regressions in the MS and NC groups (with independent variable the prenatal PTSD score and dependent variable the 3 months PTSD score) showed a greater influence of prenatal symptoms on NC group (*F*(1,86) = 33.88, *AdjR^2^* = .28, β = .53, *p* < .001) than on MS group (*F* (1,88) = 6.33, *AdjR^2^* = .58, β = .26, *p* < .01). Figure 2, in which are pictured the means of PTSD symptoms, at prenatal (T1) and at 3 months (T3), show a decrease in the MS group and a increase in the NC group.

However, at 3 months, the contribution of three clusters of PTSD symptoms (re-experiencing, avoidance, and hyperarousal symptoms) is not the same. While the re-experiencing symptoms model was not significant (*F*(3,175) = 2.027, *AdjR^2^* = .01, *p* > .05); the avoidance was significant (*F* (3,175) = 8.65, *AdjR^2^* = .11, *p* < .001), due to the predictive effect of writing condition (β = -.23; *p* < .01); and also the hyperarousal model was significant (*F*(3,175) = 25.25, *AdjR^2^* = .29, *p* < .001), due to the effect of writing condition (β = -.28; *p* < .001) and of the interaction between writing condition and prenatal symptoms (β = -.19; *p* < .01).

Table 4 shows only the key results. The main effect of MS task compared to NC task indicated that women assigned to the MS group showed lower avoidance symptoms compared to NC group. Regarding hyperarousal, the product of interaction with prenatal symptoms confirmed a significantly greater influence of prenatal symptoms on NC group (*F*(1,89) = 31.57, *AdjR^2^* = .25, β = .51, *p* < .001) than on MS group (*F*(1,86) = 2.84, *AdjR^2^* = .03, β = .20, *p* < .05).

**Table 4:** Regression Analysis Predicting Avoidance and Hyper-Arousal Scores at 96 Hours (T2) and at 3 Months (T3)

Predictor variables	Avoidance (z-scores)			Hyper-arousal (z-scores)		
	*B*	*SE B*	β	*B*	*SE B*	β
96 Hours (T2)						
*Constant*	-.02	.07		-.01	.07	
Prenatal Symptoms Scores	.25	.07	.21*	.31	.07	.31**
Writing Condition	-.04	.14	-.02	-.18	.14	-.09
Prenatal Symptoms Scores*Writing Condition	-.27	.15	-.13	-.34	.14	-.16*
3 Months (T3)						
*Constant*	-.01	.07		-.01	.06	
Prenatal Symptoms Scores	.21	.07	.21*	.39	.06	.39**
Writing Condition	-.46	.14	-.23**	-.56	.12	-.28**
Prenatal Symptoms Scores*Writing Condition	-.15	.15	-.07	-.38	.12	-.19**

*Note.* *p < .05. **p < .001.

## Discussion

The purpose of our research was to evaluate whether giving meaning to stressful experience of childbirth could moderate the relationship between antenatal and post-natal depressive and post-traumatic (PTSD total score, re-experiencing, avoidance, and hyperarousal) symptoms, both at 96 hours post-partum and at 3 months post-partum. We expected that women assigned to the “making-sense group” would show a significant reduction of depressive and post-traumatic symptoms, both at 96 hours post-partum (H1) and at 3 months post-partum (H2), while women assigned to the “control-neutral group” would have an increase in these symptoms or would not present differences compared to antenatal phase.

Our data showed a statistically significant effect for the making-sense intervention on the trajectory of depression and posttraumatic stress. Regarding depressive symptoms, the making-sense task had a predictive effect at 96 hours and a significant moderating effect at 3 months on the association between prenatal and postnatal symptoms; regarding posttraumatic symptoms, the making-sense task had a significant moderating effect only at 3 months. Therefore, our hypothesis 1 (H1) is confirmed only for depressive symptoms, while our hypothesis 2 (H2) is fully confirmed. To comment on these data, we should immediately note that the adjusted *R^2^* values for the models tested in our research, which included the making-sense task and prenatal symptoms, explained only a portion of variance between 16% and 29%. This could mean that the reduction of other risk factors, described in the introduction (for example, lack of marital and social support and stressful events), could be explicative predictors of the improvement observed. Furthermore, we cannot exclude that, parallel to the MS intervention, the women expressed to other people negative emotions connected to childbirth. However, the immediacy of our intervention, and some sentences which emerged from written protocols, lead us to consider it highly improbable that these themes had been treated before. It is equally improbable that antenatal courses (attended by a large number of women equally distributed between the MS and NC groups) were opportunities for communicating and narrating. In fact, they were essentially dedicated to physical activities (gymnastics and yoga) and also to lessons on different types of childbirth, breastfeeding, and returning home with the child, and not to investigation of post-partum emotions.

Having clarified these points, we can explain the improvement in depressive and posttraumatic symptoms which emerged from our data, considering the comorbidity between the two disorders and the fact that there are some areas of overlap (Czarnocka & Slade, 2000; Leeds & Hargreaves, 2008; Wenzel, Haugen, Jackson, & Brendle, 2005; White, Matthey, Boyd, & Barnett, 2006).

Depression shares with anxiety disorders feelings of fear for the future, sleep disturbances, and difficulties in concentration, which can entail a failing self-perception, a catastrophic vision of the world, and a hopeless perception of the future (Beck, 2004; Wijma et al., 1997). In particular, Joiner et al. (1999) noted that physiological hyperarousal is a cohesive, discriminable, and valid construct for understanding the relation of depressive and anxious syndromes; and Ayers, Harris, Sawyer, Parfitt, and Ford (2009), in a wide research study on 1,423 women assessed one year after childbirth, showed a strong correlation between depression and PTSD numbing and arousal symptoms. But also avoidance symptoms and avoidance coping perform an important role both in depression and in anxiety disturbances (Barlow, Allen, & Choate, 2004). Avoidance was identified by Leventhal (2008) as one of the fundamental mechanisms common to mental disorder, and represents the principal factor in transformation of feelings of sadness into depression. Our data confirmed that, in prenatal phase, all the women (*n* = 7) with clinical level of depression (cut-off > 20) presented a comorbidity with PTSD severity (cut-off ≥ 25.26). In addition, a strong correlation emerged, in the full group, between depression and hyperarousal (*r* = .645) symptoms and between depression and avoidance symptoms (*r* = .541). This overlap could explain why the intervention of making sense was particularly sensitive in reducing depressive and PTSD avoidance and hyperarousal symptoms, so that women assigned to this group compared to the control group showed, at 3 months, both less avoiding thoughts, feeling of detachment or estrangement from others and less irritability, inability to concentrate, fatigue, state of alarm, worry, and difficulty in falling asleep etc.

However, from our data the positive effect of making sense on PTSD symptoms was noticed at three months and not early (96 hours). These results are in line with previous studies in which the beneficial outcomes were detected from one to five weeks after writing intervention (Frattaroli, 2006; Sheese, Brown, & Graziano, 2004). Moreover, in a study by Smyth, Stone, Hurewitz, and Kaell (1999) on rheumatoid arthritis, positive changes were noted only at a distance of four months, but not at two weeks and at two months. It must be said that, more generally, Pennebaker underlines that the links between writing and PTSD are still tenuous, also due to the fact that many (perhaps most) PTSD-prone individuals will not benefit from any simple interventions (Pennebaker & Chung, 2011). Even Koopman et al. (2005), in their research of expressive writing on depression and PTSD among women surviving intimate partner violence, concluded that it is harder to ameliorate PTSD symptoms than depression.

We can hypothesize that writing about the experience of childbirth, particularly if it is stressful and characterized by ambivalent emotions, brings to light discrepant and contradictory thoughts and perceptions, which can activate avoidance mechanisms requiring time to be processed. Some sentences taken from the making-sense task are examples of emotional complexity and of consequent difficulty in elaboration.

A. wrote:

"*It was the most upsetting and unexpected experience of my life, of which I can’t talk to anyone. No matter how prepared one thinks one is, one is faced with an event for which there is no word. I wonder how it is possible that most women have undergone this experience and apparently their life appears unchanged as if it were a normal event (but, in fact it is, otherwise the world would have already stopped some time ago).*"

B. explained:

"*Childbirth is a unique experience, in every sense, for better and for worse. A pain and a suffering, fortunately, never felt before, a desire to stay alone, non to see either my husband or the child, and everybody congratulating me. I was not prepared and did not expect what I have suffered and I wondered why it happened to me and if it was happening only to me, if I had done something wrong or failed to do something.*”

The tendency to avoidance thoughts and emotions like these is not without consequences, because it blocks learning, inhibits capacity to cope with requirements of the environment and with affectively near persons, and leads to a sort of mechanical and routine attitude, which is bored, cold, worried, and unaffectionate, together with a diminution of interest and positive reactivity to stimuli. The containment and elaboration of the automatic and unconscious processes of evasion, removal, and avoidance of negative thoughts, on the contrary, represent a protective factor that contributes to a better state of psychological health (Barlow, Allen, & Choate, 2004; Kernan & Lepore, 2009). In line with these considerations, our data demonstrated the main effect of the making-sense task on avoidance symptoms (β = -.23; *p* < .001). The reduction of depressive and post-traumatic symptoms detected in mothers of our study could also be explained by considering that the data of this research concern a majority of women with sub-threshold, light or mild distress levels (*n* = 169 with depression score ≤ 19 and *n* = 166 with PTS score < 25.26) who we usually consider can improve spontaneously. This commonplace is disproved by the reminders of those studies which invite us not to underestimate the cases of sub-threshold depression (called sub-syndromal depression or minor depression), which are very prevalent in the general population and which can determine damage also for society, especially in the form of reduced productivity while at work (Furukawa et al., 2012). Symptoms which, even when they are minor or subthreshold, in a certain percentage of women could become chronic and stabilize (Furukawa et al., 2012; Reck et al., 2009). Therefore, if we try to further the analysis of our data in a clinical perspective, we note that they confirmed the positive effect of MS intervention in opposing the chronicization and worsening of symptoms. The evolution of depressive symptoms in the majority of women, examined here, with subthreshold levels of depression in the prenatal phase (*n* = 169 with depression score ≤ 19, *n* = 166 with PTS score < 25.26), indicated that none of the 86 women in the MS group presented clinical values for depression at three months, while 3 of the 83 women in the NC group were affected. Also, the trajectory of PTS in women with sub-threshold values (*n* = 166 with PTS score < 25.26) showed that 1 out of 85 of the MS group presented clinical values for PTS at three months, against 5 women out of 81 in NC group. Encouraging results also emerge from the small subgroup of women with clinical depression scores (*n* = 7 with moderate-severe depression, cut-off ≥ 20) and PTS (*n* = 10 with PTS score > 25.26) in the prenatal phase. At three months from childbirth, depressive symptoms remained clinically significant (*M* = 21.50) in the three women of the NC group, while they decreased noticeably (*M* = 10.12) in the four women of MS group. Similarly for PTS, none of the four women of MS group presented clinical levels of PTS, while 4 out of 6 women in NC group continued to present clinically significant PTS scores (PPQ: cut-off > 19).

These clinical results, even though limited and partial, encourage us to think that universal preventive interventions, specifically if based on narration and attribution of coherent meanings to ambivalent or negative experiences, may be effective both in improvement of well-being of women with light levels of distress and in interception of critical situations which could become chronic.

### Limitations and Strengths

The methodological limitations of this study, however, lead us to consider carefully the results and interpretations derived from it. A first limit of the research, already mentioned above, consists in the fact that the effects of risk factors, pertinent to this type of study, like social support by the family or by health service staff, and, in particular the type and level of interpersonal communication after childbirth, have not been verified. Even if we consider that there have not been interference factors, it is important, in a later study, to verify better the effect of these risk factors also through administering a brief questionnaire to the women to understand if it received support, which type and from whom. This is also to monitor whether the support network in a natural setting favoured, in a natural path of exchange and communication, the narration of the negative aspects connected with childbirth that we introduced in our intervention

A second and important limitation is the methodology employed here that theoretically relying on the Model of Meaning Making has not then thoroughly analysed the content of the writing task with the appropriate categories. Although the principal purpose of this study was to examine the efficacy of the making-sense intervention, it would have been useful – similarly to the methodology employed by Boals (2012) in a study on undergraduate students invited to write about negative autobiographical events – to define an evaluation grid to analyse specifically the degree of meaning making (no search for meaning-making, little, moderate, or active search for meaning-making) adopted by new mothers in distress elaboration. A third limit consists in the number of writing sessions which in this study was limited to one instead of three distanced from each other, necessary to obtain a greater efficacy of this protocol (Frattaroli, 2006), above all in the case of PTSD symptoms which are more resistant to change than depressive ones. The reasons for this choice have been explained above and are coherent with the purposes of the current research. Finally a further methodological doubt arises from some considerations by Solano et al. (2007), who decided in their research not to have the patients in the control group waiting for a surgical intervention write about “neutral” or “trivial” topics. The authors maintain that for a person staying in the hospital waiting for an operation, it would be very difficult to find really “neutral” topics. The same problem emerged in our research, in the case of the new mothers of the control group, who sometimes did not really describe a typical and neutral day but reported the day of the birth of their child. It could be opportune in further studies to introduce a second control group with no performance of the making-sense task. A last limit of this study concerns the under-representation of women who are not partnered or from different cultures and, for these reasons, these results may have limited generalizability. Furthermore all data collected in the study was based on self-reported information, and thus it prevented a deeper verification by many information sources.

In spite of these limits we consider that writing a narrative about post-partum experience, adopted in this study for the first time, may be a helpful intervention, particularly in the immediacy of childbirth to allow this delicate transition phase to enter into the autobiographical life story in a coherent and unitary way (McAdams, 2001). Also, in training for nurses who attend new mothers or in usual hospital practice it could be appropriate to train staff on the potential of the making-sense task, which permits free communication on themes and subjects which women could be reluctant to discuss verbally. We cannot exclude that even in the childbirth preparation courses, at present dedicated above all to the practical and concrete aspects, the use of written narrative may constitute a method of self-help to communicate to women to channel their attention onto the deepest psychological dimensions of stressful experiences.
